# Prostate-specific antigen rising in Iranian men in correlation with body mass index, fasting blood sugar and blood lipid profile

**DOI:** 10.15171/jnp.2016.25

**Published:** 2016-08-07

**Authors:** Davood Arab, Arash Ardestani Zadeh, Majid Mirmohammadkhani, Azadeh Beiglarzadeh

**Affiliations:** ^1^Department of Surgery, Clinical Research Development Center, Semnan University of Medical Sciences, Semnan, Iran; ^2^Social Determinants of Health Research Center, Kowsar Hospital, Semnan University of Medical Sciences, Semnan, Iran

**Keywords:** Prostate-specific antigen, Triglycerides, Cholesterol, Fasting blood sugar, Body mass index

## Abstract

**Background:**

Prostate-specific antigen (PSA) is a serine protease that is secreted by prostate cells and it is useful as a tumor marker for prostate cancer.

**Objectives:**

In this study, the relationship between some of metabolic factors and serum PSA level was investigated.

**Materials and Methods:**

In this cross-sectional study, patients with urinary symptoms or for screening of the prostate cancer (after 50 years of age or 40 years with a family history of prostate cancer), were evaluated. Collected data included metabolic syndrome factors such as cholesterol (Chol), triglycerides (TG), fasting blood sugar (FBS), and body mass index (BMI), serum PSA level, prostate volume and age.

**Results:**

481 patients were enrolled to this study with the average age of 60.69 ± 9.72 years and the average PSA level of 1.70 ng/ml. Data analysis showed that there was a significant relationship between serum PSA level with age (*P* < 0.001, r = 0.30) and prostate volume (*P* < 0.001, r = 0.29). There were not significant relationship between serum PSA level with TG (*P* = 0.57, r = 0.026), Chol (*P* = 0.57, r = -0.025), FBS (*P* = 0.054, r = 0.088), and BMI (*P* = 0.89, r = 0.006).

**Conclusions:**

This study showed that, with increasing age and prostate volume, serum PSA level increased, and an increase in the levels of cholesterol, TG, FBS and BMI did not have significant effect on serum PSA level.

**Implication for health policy/practice/research/medical education:**

Prostate-specific antigen (PSA) is a serine protease that is secreted by prostate cells and it is useful as a tumor marker for prostate cancer. Screening by PSA, has reduced the age of the detection of prostate cancer and it has become a middle-aged people cancer. Data analysis showed that there was a significant relationship between serum PSA level, age and prostate volume and there were not a significant relationship between serum PSA level, TG, Chol, FBS and BMI. This study shows that, with increasing age and prostate volume , serum prostate-specific antigen (PSA) level increased, and an increase in the levels of cholesterol, triglycerides (TG), fasting blood sugar (FBS) and body mass index (BMI) did not have significant effect on serum PSA level.

## 1. Background

Prostate cancer is the fourth prevalent cancer in the world and the most prevalent non-skin malignant disease in men, and the second cause of cancer death in American men, and its prevalence is higher in developing countries (1). Screening by prostate-specific antigen (PSA), has reduced the age of the detection of prostate cancer and it has become a middle-aged people cancer (1).

PSA (human kallikrein 3) is a serine protease, and it is almost exclusively secreted by prostate epithelial cells, and it is used to detect prostate cancer in asymptomatic individuals over 50 years, people over 40 years with a family history of prostate cancer, and those who come with prostatism symptoms (2).

Even though serum PSA level in men is considered less than 4 ng/ml, but this amount is influenced by various factors such as benign prostatic hyperplasia, a patient’s age, prostate weight, recent history of prostate massage or manipulation and severe infection of the prostate or prostatitis. In cases that the PSA is between 4-10 ng/ml, the use of other tests such as PSA density (PSA to prostate weight ratio), PSA velocity (the increase in PSA over a year), and the ratio of free PSA to total PSA is helpful to distinguish prostate cancer from other prostatic diseases (1).

PSA test is not a highly specific test to diagnose prostate cancer, because in 28% of cases, people affected by prostate cancer may have a PSA less than 4 ng/ml (2).

Thus, considering the prevalence of prostate cancer and the importance of PSA in detecting prostate cancer, several studies have been examined, in relation to PSA level in different people and its relation to other factors that the most important of them was the relationship between PSA and a number of metabolic syndrome components such as high blood sugar, high blood lipids and obesity (3-11). Since the liver is the main place for metabolism and cleaning of PSA, so the fatty liver disease in obese people can affect the metabolism of PSA (8,11).

On the other hand, obese men may have lower PSA levels due to the high plasma volume and as a result diluted blood (12), and it is also thought that the lipid levels can affect the serum PSA levels through the testosterone metabolism (8). In most studies, there has been a relationship between these components and PSA level, for instance, some limited studies have shown that serum PSA level in men affected by diabetes is lower than healthy people, due to the testosterone level (8,11). In some other studies, PSA in obese people with high body mass indexes (BMIs) was less than that in normal people (13-18).

## 2. Objectives

Since researches on the biochemistry of PSA, have a high influence on the potential for cooperation between the discovery and management of prostate cancer, and considering the high prevalence of metabolic syndrome and risk of metabolic syndrome impact on the PSA, as well as the lack of similar studies in Iran and reference books, we decided to study the impact of BMI, blood sugar and blood lipid profile on serum PSA level.

## 3. Patients and Methods

### 3.1. Study population

In this cross-sectional study, the study population were all of eligible patients who came to the urology clinic of Kowsar hospital in Semnan from May 2013 to April 2014, because of urinary symptoms (i.e. prostatism) or for screening of the prostate cancer, after 50 years of age or 40 years with a family history of prostate cancer.

Inclusion criteria included patients with benign prostate disorders that were proved by PSA and rectal examination, whether the patient had came for screening or was a candidate for medical or surgical treatment. If the patient had urinary tract infection, he underwent antibiotic treatment and PSA testing was performed again. If the PSA was between 4-10 ng/ml, the patient was asked to perform the free to total PSA test again. Patients who had a history of prostate cancer at present or in the past and were under supervision or treatment, and also patients for whom there was a strong suspicion of prostate cancer due to PSA greater than 10 or by abnormal free to total PSA test (in case of PSA being between 4-10 ng/ml), or abnormal prostate consistency in rectal examination, and he was a candidate for prostate biopsy, were excluded from the study.

A questionnaire was used to collect the information. The patients age, height and weight and size of the prostate through the rectal examination and sonography, were recorded. Also all patients were asked to do PSA, fasting blood sugar (FBS), and blood lipid tests including cholesterol and triglycerides (TG) and the results were recorded.

### 3.2. Ethical issues

1) The research followed the tenets of the Declaration of Helsinki; 2) informed consent was obtained, and they were free to leave the study at any time; and 3) the research was approved by the ethical committee of Semnan University of Medical Sciences.

### 3.3. Statistical analysis

To show magnitude and direction of probable associations, we calculated Spearman’s correlation coefficient using SPSS 16 software, and P value less than 0.05 was considered as significant.

## 4. Results

In this study data of 481 people were analyzed. The mean age of the participants was 60.69 ± 9.72 (SD), the youngest was 36 years old and the oldest was 97 years old. Among all participants, 243 ones (50.5%) were 60 years old and younger, and 238 ones (49.5%) were older than 60. Table 1, the frequency distribution of the participants in terms of the variables of the study, separately shows their values.

**Table 1:** The frequency distribution of the participants by the variables of the study.

**Specifications**		**Number**	**%**
Age group	60 years and younger	243	50.5
	> 60 years	238	49.5
PSA levels	4 and less	443	92.1
	> 4	38	7.9
BMI	25 and less	155	32.2
	> 25	326	67.8
Chol	200 and less	352	73.2
	> 200	129	26.8
FBS	110 and less	332	69
	> 110	149	31
TG	150 and less	231	48
	> 150	250	52

In terms of the checked PSA values, mean PSA value for these people was 1.70 ± 1.44 (SD), ng/ml. Table 2 shows the value of cholesterol, TG, FBS, prostate volume and body mass index (BMI).

**Table 2:** Mean and standard deviation values of the prostate volume, body mass index, FBS and blood lipid profile

**Index**	**Mean**	**Standard deviation**
Chol	178.42	39.89
TG	168.57	82.39
FBS	112.87	45.43
Prostate Volume	37.89	13.44
BMI	27.44	4.90

Considering the correlation coefficient between laboratory factors related to metabolic syndrome and PSA, there was a significant positive correlation between PSA and age, revealed the PSA level increased along with age (*P*<0.001, r=0.3). Data analysis also showed, a significant relationship between serum PSA level, and prostate volume (*P*<0.001, r=0.29). However no significant relationship between serum PSA level and TG (*P*=0.57, r=0.026), FBS (*P*=0.054, r=0.088), Chol (*P*=0.57, r=-0.025) and BMI (*P*=0.89, r=0.006) were observed. Table 3 shows this relationship.

**Table 3:** Correlation coefficient between age, prostate volume and the metabolic indexes (body mass index, FBS and blood lipid profile) and PSA

**Index**	**Correlation coefficient**	**P value**
Age	0.30	< 0.001
Chol	-0.025	0.57
TG	0.026	0.57
FBS	0.088	0.054
Prostate volume	0.29	< 0.001
BMI	0.006	0.89

## 5. Discussion

PSA is a glycoprotein that is secreted primarily from prostate cylindrical cells, and is strongly influenced by androgens. Even though it is organ-specific but it is not cancer-specific. There is a considerable overlap in PSA levels among men with benign prostate diseases versus men with malignant prostate disease (1,2). Some of the factors which have been proven to affect serum PSA level include age, race, and prostate volume. Prostatitis (acute and chronic) and urinary retention can increase PSA to different levels. Biopsy and digital rectal examination can rise the PSA level and cause a false positive result. Ejaculation can also increase PSA level that result in a false positive and up to 48 hours it can return to baseline (1,2). Also in recent years, studies have been investigated in relation to PSA levels in different people and its relation to other factors which the most important of them was the relationship between PSA and a number of metabolic syndrome components (high blood sugar, high blood lipids, and obesity) (3,11). For instance a study which was performed in Korea by Yang et al on healthy men who had referred for checkup, showed that serum PSA level has inverse relation with BMI in people with high weight (BMI > 23 kg/m^2^) in 40-59 years of age, and the reason of this relationship has been explained with these two hypotheses:

1- PSA concentration is regulated by androgens, and androgenic activities decrease in obese people.
2- People with high BMI have high plasma volumes which can decrease serum PSA concentration (10).

Another study by Liu-Ming et al on healthy men who went to urologic clinics for check-ups had the same result (9).

In a study that was conducted by Han et al about the relationship between some metabolic syndrome factors such as FBS, TG, high-density lipid (HDL-C), BMI, age and serum PSA level (8), it was shown that in people with high BMI, sex hormone binding globulin (SHBG) levels decrease, and SHBG acts as a testosterone binding protein in plasma. The conversion of testosterone to estradiol can increase in obesity conditions, but SHBG production is typically associated with obesity inversely. The findings suggest that obesity can be a cause of hypogonadism through the conversion of testosterone to estradiol in adipose tissue and decrease SHBG production. Similarly, in men with a high BMI, serum PSA levels can decrease due to the blood dilution. HDL-C affects serum PSA levels through testosterone levels. In healthy men a decrease in endogenous testosterone levels are associated with an increase in TG and decrease in HDL-C, which have negative effects on PSA levels. PSA levels are regulated by androgens, and testosterone levels are lower than normal in men with type 2 diabetes, and it can be due to a decrease in SHBG, and there is a negative relationship between insulin resistance and serum PSA levels. The results of this study proved a certain relationship between age and serum PSA levels. Similarly, in relation to FBS, in a study by Han et al, it is found that serum PSA levels in diabetic people are lower than non-diabetic people, and that is because total testosterone levels in people with type II diabetes are lower than that in normal people, and this difference could be due to low SHBG (8).

Also the study, conducted by Mohammad et al, showed a negative relationship between serum PSA level and BMI, TG, and FBS, with similar reasons (19).

In another study that was conducted by Cvitkovic et al, a negative relationship between FBS and serum PSA level, as well as a positive relationship between age and PSA level was shown. The cause of a positive relationship between PSA and age is that prostate volume increases with age, thus the production content of PSA in prostate tissue increases (20). Moreover, a study, conducted by Yang et al, showed that there was a positive relationship between central obesity and prostate volume, also there was a negative relationship between serum PSA level and an increase in BMI (7). Accordingly, in a study, conducted by Ahn et al, a negative relationship between serum PSA level and BMI was observed, and age in this group which includes 20-39 years people, did not show a relationship with serum PSA level (6). Another study, by Jeong et al, showed that serum PSA level in Asians can be different from that in Westerners (5). Our study showed a positive relationship between PSA level, and age as well as the volume of prostate, and also there was no relationship between PSA and Chol, TG, FBS and BMI, so that actually no relationship with an effect on PSA levels was observed by increasing each variable listed above.

There were limitations to this study, as in other studies, which can somewhat affect the results of the study, including the limited population, the type of diabetes and received medicine in study subjects had not been separated. In relation to high BMI, the type of diet may also affect the results, as well above all is that the absence of prostate cancer in men was not proved by biopsy.

## 6. Conclusions

The present study showed that serum PSA level increases with age, and prostate volume, while no relationship were observed to changes in PSA levels, by increasing TG, Chol, FBS and BMI. We hope that, in the near future, we can increase the precision of the study by increasing statistical population and reducing the restrictions which can be somewhat effective on clinical judgment.

## Limitations of the study

This is a single-center non-randomized study with a limited number of patients.

## Acknowledgments

This study is adapted from the M.D. thesis of Azadeh Beiglarzadeh (Thesis#417). We gratefully thank the Research and Technology Deputy Semnan University of Medical Sciences and all the patients and people who assisted us in conducting this thesis. We would like to thank the Clinical Research Development Unit of Kowsar Educational and Research and Therapeutic Center of Semnan University of Medical Sciences for providing facilities to this work.

## Conflicts of interest

The authors declared no competing interests.

## Authors’ contribution

DA, AA and AB conducted the research. MM analyzed the data. AB prepared the primary draft. DA and AA edited the final draft. All authors signed the manuscript.

## Funding/Support

This research was supported by Surgery Department, Semnan University of Medical Sciences, Semnan, Iran.
